# Selective apoptosis induction in MCF-7 cell line by truncated minimal functional region of Apoptin

**DOI:** 10.1186/1471-2407-13-488

**Published:** 2013-10-21

**Authors:** Lim Shen Ni, Zeenathul Nazariah bt Allaudin, Mohd Azmi b Mohd Lila, Abas Mazni b Othman, Fauziah bt Othman

**Affiliations:** 1 Institute of Biosciences, Universiti Putra, Serdang, Malaysia; 2 Faculty of Veterinary Medicine, Universiti Putra Malaysia, Serdang, Selangor 43400 UPM, Malaysia; 3 Institute of Agrobiotechnology, Serdang, Malaysia; 4 Faculty of Medicine, Universiti Putra, Serdang, Malaysia

**Keywords:** VP3, Apoptin, MCF7 cells, Chang cells, Apoptosis, Microinjection, Truncation

## Abstract

**Background:**

Chicken Anemia Virus (CAV) VP3 protein (also known as Apoptin), a basic and proline-rich protein has a unique capability in inducing apoptosis in cancer cells but not in normal cells. Five truncated Apoptin proteins were analyzed to determine their selective ability to migrate into the nucleus of human breast adenocarcinoma MCF-7 cells for inducing apoptosis.

**Methods:**

For identification of the minimal selective domain for apoptosis, the wild-type Apoptin gene had been reconstructed by PCR to generate segmental deletions at the N’ terminal and linked with nuclear localization sites (NLS1 and NLS2). All the constructs were fused with maltose-binding protein gene and individually expressed by *in vitro* Rapid Translation System. Standardized dose of proteins were delivered into human breast adenocarcinoma MCF-7 cells and control human liver Chang cells by cytoplasmic microinjection, and subsequently observed for selective apoptosis effect.

**Results:**

Three of the truncated Apoptin proteins with N-terminal deletions spanning amino acid 32–83 retained the cancer selective nature of wild-type Apoptin. The proteins were successfully translocated to the nucleus of MCF-7 cells initiating apoptosis, whereas non-toxic cytoplasmic retention was observed in normal Chang cells. Whilst these truncated proteins retained the tumour-specific death effector ability, the specificity for MCF-7 cells was lost in two other truncated proteins that harbor deletions at amino acid 1–31. The detection of apoptosing normal Chang cells and MCF-7 cells upon cytoplasmic microinjection of these proteins implicated a loss in Apoptin’s signature targeting activity.

**Conclusions:**

Therefore, the critical stretch spanning amino acid 1–31 at the upstream of a known hydrophobic leucine-rich stretch (LRS) was strongly suggested as one of the prerequisite region in Apoptin for cancer targeting. Identification of this selective domain provides a platform for developing small targets to facilitating carrier-mediated-transport across cellular membrane, simultaneously promoting protein delivery for selective and effective breast cancer therapy.

## Background

In the new era of biotechnology, the study in mass production of recombinant protein has attracted a lot of attention towards protein therapy which can readily overcome the limitations faced in gene therapy and plasmid DNA-based vaccines. The genetic elements of plasmid as a propagation and expression units as well as their host genome for the production of recombinant plasmid DNA are still a safety concern for clinical use [1,2]. On the other hand, protein therapy can overcome this problem by being highly specific in action with less potential to interfere with normal biological process and without the need to be activated for gene expression [3].

Apoptin is a small protein of 121 amino acids (a.a.) derived from chicken anemia virus (CAV) (Noteborn *et al.*, [4]) and possess tumor-specific apoptosis-inducing activity. In tumor cells, it predominantly co-localizes with heterochromatin and nucleoli, followed by induction of apoptosis in various cultured human tumorigenic and/or transformed cell lines such as breast and lung tumor, leukemia, lymphoma, osteosarcoma melanoma, cholangiocarcinoma, hepatoma and bladder cancer cells [5,6].

Apoptin is predominantly found in the nucleus of transformed and tumor cells, whereas in untransformed cells, it localizes the cytoplasm. The a.a. sequence of Apoptin contains two basic stretches constituting putative nuclear localization signals (NLS) and a stretch of nuclear export signal (NES) [7]. The nuclear localization regions contain a bipartite-type NLS (a.a. 82–88 and 111–121) and the distinct parts of Apoptin have independent, intrinsic cell killing activity strongly correlated with induction of cell death (Danen-van *et al.,*[8]). Whilst, NES residues at a.a. 97–105 enable the Apoptin transportation from the nucleus to cell cytoplasm [7]. Apoptin also comprises of a hydrophobic leucine-rich stretch (LRS) at a.a. 33–46 which assists in Apoptin nuclear accumulation that act as nuclear retention sequence [9].

Although popularly investigated, the mechanism of Apoptin’s tumor-specific apoptosis activity remains to be fully elucidated. The protein becomes specifically phosphorylated and activated by a kinase activity found only in tumor or transformed cells. Apart from nuclear targeting signals, phosphorylation site at threonine-108 (Thr-108) is pivotal in transducing apoptotic signals in cancerous cells [10]. However, some findings reported the partial influence of Thr-108 phosphorylation in apoptotic activity [11,12]. Recent finding by Lanz indicated the role of proteasome in Apoptin degradation but had no influence on the amount of Apoptin in tumor cells, although it affects p53 level. Normal cells stabilize the Apoptin and p53 levels [13]. On other hand, Yuan L. *et al*. demonstrated Apoptin bound to heat shock protein 70 (HSP70), an anti-apoptotic protein, causing downregulation of HSP70 expression and subsequent apoptosis induction in tumor cells. Apoptin has no effect on HSP70 that was not activated in normal cells. Thus, this study explained why apoptotic effect occursin tumor cells only and not in normal cells [14].

The main aims of this study are i) to determine the minimal functional site of Apoptin, ii) to assess the contribution of LRS in the absence of NES, iii) to analyze the early cytoplasmic events in the subcellular trafficking of truncated Apoptins and iv) to explore the feasibility of direct delivery of truncated Apoptins as antitumor agents. The recombinant truncated Apoptin proteins that were fused with maltose-binding protein (MBP) at their N-terminus were produced by *in vitro* protein expression system. Maltose binding protein (MBP) fused Apoptin exists as a stable, homogenous globular multimeric complex, which presents the active form of Apoptin *in vivo*[15]. MBP causes neither toxicity nor apoptosis of cells. Prokaryotically expressed recombinant MBP-Apoptin protein maintains its tumor-specific killing and localization properties, indicating that a mammalian-specific folding environment is not a necessity [16]. In this study, we hypothesize that the presence of minimal selective domain in truncated Apoptin complexes directs the apoptotic induction in cancerous cells.

## Methods

### Construction of truncated MBP-apoptin plasmids

**Figure 1:**
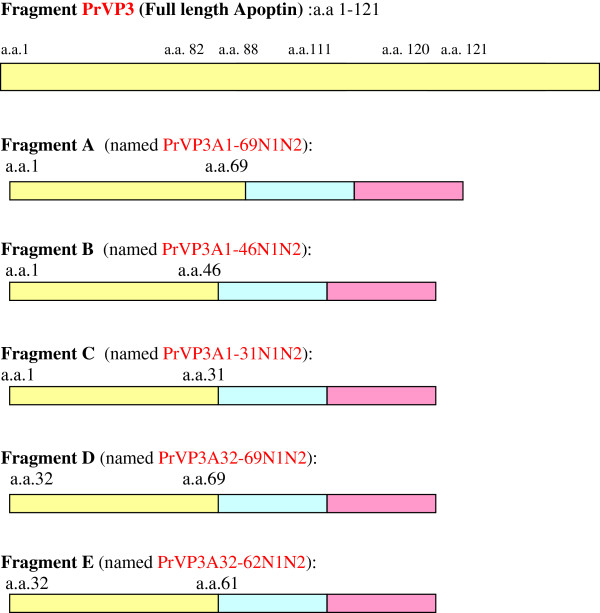
**Diagrammatic design of Apoptin (PrVP3) and truncated Apoptins.** (PrVP3A1-69N1N2, PrVP3A1-46N1N2, PrVP3A1-31N1N2, PrVP3A32-69N1N2, PrVP3A32-62N1N2).

Plasmids-pIVEX-MBP (Roche Diagnostic, Germany) was used for the expression of proteins as fusions to the C-terminus of MBP (maltose binding protein). PCR was conducted to amplify 5 sets of DNA fragment Apoptin (Patent: PI20100031130); VP3A1-69N1N2, VP3A1-46N1N2, VP3A1-31N1N2, VP3A32-69N1N2 and VP3A32-62N1N2. Nine sets of primers were used to amplify the DNA fragments according to the designated truncation (Figure 1). The DNA fragments were then digested with *Nco*1 and *Xho*1 and ligated into pIVEX-MBP vectors at the C terminus of MBP with the appropriate linkers in between Nco1 and Xho1, generating pMBP-VP3 (a.a 1–121), pMBP-VP3A1-69N1N2 (a.a 1–69 with NLS1 and NLS2), pMBP-VP3A1-46N1N2 (a.a 1–46 with NLS1 and NLS2), pMBP-VP3A1-31N1N2 (a.a. 1–31 with NLS1 and NLS2), pMBP-VP3A32-69N1N2 (a.a. 32–69 with NLS1 and NLS2) and pMBP-VP3A32-62N1N2 (a.a. 32–62 with NLS1 and NLS2) (Figure 1).

### Protein expression

Proteins of the constructs pMBP-VP3, pMBP-VP3A1-69N1N2, pMBP-VP3A1-46N1N2, pMBP-VP3A1-31N1N2, pMBP-VP3A32-69N1N2 and pMBP-VP3A32-62N1N2 were expressed by using *in vitro* protein synthesis system, Rapid Translation System (RTS) (5 Prime, Germany) according to manufacturer’s instruction. Briefly, RTS uses a cell-free protein production system by utilizing an enhanced *E. coli* lysate to perform coupled *in vitro* transcription-translation reactions.

### Western blot assay

The expressed fusion proteins were separated by SDS PAGE and confirmed via Western blot assay by using anti-VP3 monoclonal antibody (mAb) (TropBio, Australia) and anti-MBP mAb (New England Biolabs, USA).

### Protein purification

The expressed proteins were purified by amylose resin (New England Biolabs, USA). 300 μl of amylose resin was added into purification column. The column was equilibrated with 8 column volumes equilibration buffer (10 mM Tris–HCl, pH 7.2). The expressed protein was applied into the column by gravity flow. The column flow was shut off after the entire sample has entered the column. The column was incubated for 30 min. The column was washed with 10 bed volumes of wash buffer (10 mM Tris–HCl, 1 M NaCl, pH 7.2). The protein was eluted with 4 bed volumes of elution buffer (10 mM Tris–HCl, 20 mM maltose, pH 7.2). The eluted protein was concentrated using Vivaspin 2 Concentrator (Vivascience, Germany).

### Cells

Experiments were performed onMCF-7 (provided by Professor Fauziah Othman, Faculty of Medicine, University Putra Malaysia) and non-cancerous human liver cell (Chang’s cell) (provided by Associate Professor DrZeenathul Nazariah Allaudin, Faculty of Veterinary, University Putra Malaysia. The maintenance medium for both cell lines contained RPMI supplemented with 10% fetal bovine serum (FBS) and an antibiotic solution (100 units/ml penicillin, 100 μg/ml streptomycin). All cell culture media and supplements were from Gibco Invitrogen Life Sciences (Paisley, UK). Both MCF-7 cells and Chang cells were plated at a density of 2×10^3^ onto a cover slip with size 22×22 mm^2^ 24 h prior to microinjection.

### Microinjection

Apoptin protein was delivered into MCF-7 cells and Chang cells through microinjection. Optimization of cellular microinjection has been previously established in our laboratory by using MBP protein (Lim *et al*., [17]). 6 μg of protein was loaded into Femtotips I (Eppendorf, USA) by microloader and injected into the targeted cells under an injection pressure ranging from 30–110 hPa for 0.5 s. A total number of 50 cells per cover slip were injected.

After microinjection, the injected cells were immersed in rich RPMI 1640 media which contained 10% FBS serum and incubated at 37°C in 5% CO_2_ incubator. The injected cells were incubated at various time points; 2 hr and 10 hr prior to immunofluorescence assay and 5 hr and 15 hr before the apoptosis assay.

### Immunofluorescence

The microinjected cells were fixed after incubation of injected cells at various time points at 2 hr and 10 hr. At different time points, the cells were fixed by immersing the cover slip in 100% ice-colded methanol for 20 min at 0°C and washed thrice with PBS for 5 min each. The cover slip was then immersed in 2 ml anti-MBP mouse monoclonal antibody (Chemicon, USA) at dilution 1:1000 in 10 mM PBST buffer (3.2 mM Na_2_HPO_4_, 0.5 mM KH_2_PO_4_, 1.3 mMKCl, 135 mM NaCl, 0.05% Tween 20, pH 7.4) at RT for an hour. The cover slip was given again PBST buffer wash for 3 times at 5 min each. Two ml of goat anti-mouse IgG fluorescein conjugated secondary antibody at dilution of 1:200 in 1M PBST was used for 1 hr at RT. Subsequently, 3 PBST washes were done for 5 min each. The FITC stained cells were mounted with anti-fade agent and analyzed under fluorescence microscopy (Zeiss, USA) and the images were captured by digital image analysis equipment.

### Apoptosis assay

Injected cells were incubated with Annexin V reagent, ApopNexin FITC Apoptosis Detection kit (Chemicon, USA) to detect the appearance of the apoptotic cells. At various time points of incubation, the supernatant in the 35 mm petri dish that contained detached cells was collected into a microtube and spun down at 400×g for 5 min at 4°C. The supernatant was discarded and the pelleted cells were collected. The collected cells were resuspended with 200 μl 1X binding buffer and spun again at 400×g for 5 min at 4°C to collect the cells. This step was repeated twice to wash the detached cells. Meanwhile, those cells attached at the cover slip were washed twice with 200 μl 1X binding buffer. After the washing step, 200 μl of 1X binding buffer and 3 μl of ApopNexin FITC were added into the collected cells and mixed well. The mixture was then transferred onto the washed coverslip and incubated for 15 min at RT. After 15 min of incubation, 2 μl of counterstain, PI (Chemicon, USA) was added onto the coverslip equally and incubated again for 15 min on ice. Finally, the coverslip that contained attached and detached (flowing) cells were mounted with anti-fade agent, viewed under fluorescence microscope (Zeiss, USA) and the image captures were done by digital image analyzer.

## Results

### Construction of truncated apoptin gene

**Figure 2:**
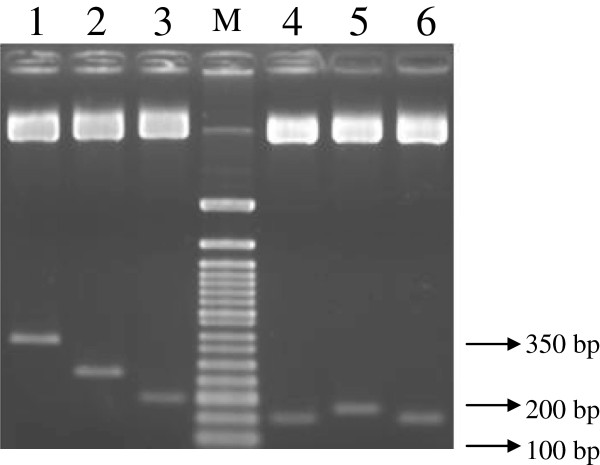
**Double digestion analysis of constructed Apoptin and truncated Apoptin.** The insert in plasmids were analysed for the presence of the truncated inserts by double digestion with NcoI and XhoI. Lane 1: wild-type VP3 gene with length 366 bp, lane 2: truncated VP3 gene pMBP-VP3A1-69N1N2 with length 261 bp, lane 3: truncated VP3 gene pMBP-VP3A1-46N1N2 with length 191 bp, lane 4: truncated VP3 gene pMBP-VP3A1-31N1N2 with length 150 bp, Lane 5: truncated VP3 gene pMBP-VP3A32-69N1N2 with length 174 bp and lane 6: truncated VP3 gene pMBP-VP3A32-62N1N2 with length 150 bp. Lane M: 50 bp DNA Ladder (New England Biolabs, USA).

Five truncated Apoptin fragments were amplified and constructed in pIVEX-MBP vector. The plasmids expressing MBP and fusions of MBP and parts of Apoptin protein have been generated. All the recombinant proteins have MBP at the N terminus of Apoptin protein. The constructed genes were named i) pMBP-VP3A1-69N1N2 (a.a 1–69 with NLS1 and NLS2), ii) pMBP-VP3A1-46N1N2 (a.a 1–46 with NLS1 and NLS2), iii) pMBP-VP3A1-31N1N2 (a.a. 1–31 with NLS1 and NLS2), iv) pMBP-VP3A32-69N1N2 (a.a. 32–69 with NLS1 and NLS2), v) pMBP-VP3A32-62N1N2 (a.a. 32–62 with NLS1 and NLS2) with lengths 261 bp, 191 bp, 150 bp, 174 bp and 150 bp respectively (Figure 2).

### Determine translocation of truncated VP3 protein

**Figure 3:**
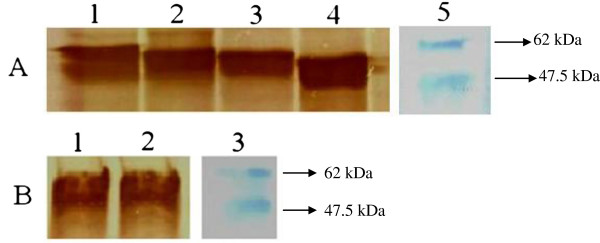
***In vitro *protein expression of Apoptin and truncated proteins.** The expressed proteins were analyzed through Western blot assay by anti-VP3 and anti-MBP mAbs. The expressed proteins were tagged with MBP, thus showed higher molecular weight (MW) instead of the original MW of wild-type Apoptin. The MW of Apoptin is 13.6 kDa. The MW of wild-type full length Apoptin and truncated Apoptin proteins tagged MBP varies from 47.5-62 kDa. Lane A1: PrVP3, lane A2: PrVP3A1-69N1N2, lane A3: PrVP3A1-46N1N2 and lane A4: PrVP3A1-31N1N2 were analysed by anti-VP3 mAb. Lane B1: PrVP3A32-69N1N2 and lane B2: PrVP3A32-62N1N2 were analyzed by anti-MBP mAb. Lane A5 and B3: Protein Marker (NEB, UK).

Proteins of interest were successfully expressed using RTS kit (5 Prime, Germany) within 6 hr of incubation and purified by amylose resin. Western blot assay verified the presence of protein of interest (Figure 3A). The findings showed positive expression for full-length and truncated Apoptin proteins. Apoptin, MBP protein (PrMBP) and its truncated proteins (PrVP3A1-69N1N2, PrVP3A1-46N1N2 and PrVP3A1-31N1N2 were analyzed by anti-VP3 mAb. All except for PrVP3A32-69N1N2 and PrVP3A32-62N1N2 proteins could be detected using this mAb (Figure 3). PrVP3A32-69N1N2 and PrVP3A32-62N1N2 can only be detected using anti-MBP mAb (Figure 3). The desired proteins from truncated constructs were expressed efficiently as well as rapidly by using *in vitro* protein expression system.

### Detection of apoptin and truncated apoptin proteins in injected normal and cancerous cells

**Figure 4:**
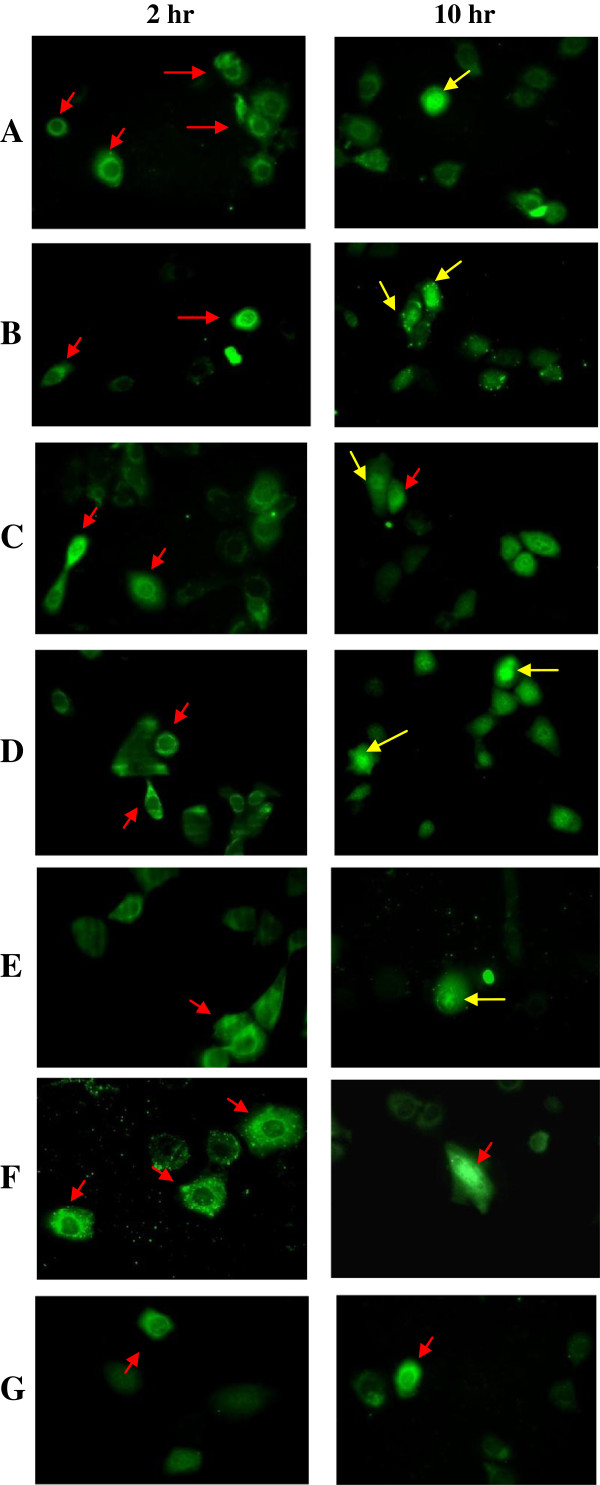
**Indirect immunofluorescence assay on microinjected MCF7 cells with VP3 protein and truncated VP3 protein.** The MCF7 cells were injected with **A)** PrVP3, **B)** PrVP3A1-69N1N2, **C)** PrVP3A1-46N1N2, **D)** PrVP3A1-31N1N2 **E)** PrVP3A32-69N1N2, **F)** PrVP3A32-62N1N2 and negative control, **G)** PrMBP. The microinjected cells were assayed at 2 hr and 10 hr. The green fluorescence showed the present of injected protein. At 2 hr, the injected protein was detected in the cytoplasm of the cells (showed in red arrow), whereas the protein was found in the nucleus of the cells at 10 hr (showed in yellow arrow) except PrMBP **(G)**.

**Figure 5:**
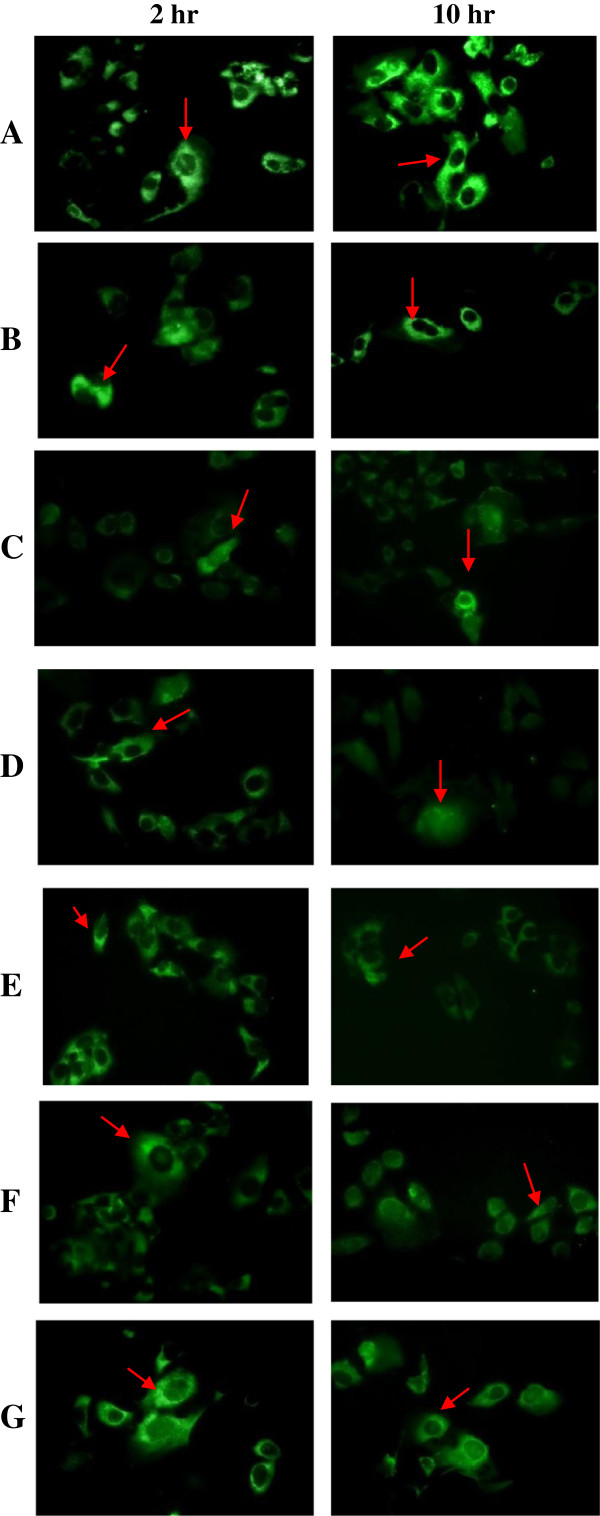
**Indirect immunofluorescence assay on microinjected Chang cells with VP3 protein and truncated VP3 protein.** Chang cells were injected with **A)** PrVP3, **B)** PrVP3A1-69N1N2, **C)** PrVP3A1-46N1N2, **D)** PrVP3A1-31N1N2 **E)** PrVP3A32-69N1N2, **F)** PrVP3A32-62N1N2 and negative control, **G)** PrMBP. The microinjected cells were assayed at 2 hr and 10 hr. At both time points, the injected protein (with green fluorescence) was detected at the cell cytoplasm (shown in red arrow).

Seven purified recombinant proteins including wild-type Apoptin (PrVP3),and truncated Apoptin proteins, PrVP3A1-69N1N2, PrVP3A1-46N1N2, PrVP3A1-31N1N2, PrVP3A32-69N1N2, PrVP3A32-62N1N2 and negative control, PrMBP were successfully delivered into MCF7 and Chang cell respectively. The cells that were successfully microinjected with proteins were shown in Figure 4 and Figure 5. All the proteins were successfully delivered into the cytoplasm of MCF7. Nuclear translocation was observed in cells microinjected with wild-type Apoptin (PrVP3) and truncated Apoptin proteins, PrVP3A1-69N1N2, PrVP3A1-46N1N2, PrVP3A1-31N1N2, PrVP3A32-69N1N2 and PrVP3A32-62N1N2. In non-cancerous Chang cells, Apoptin proteins (PrVP3), PrVP3A1-69N1N2, PrVP3A1-46N1N2, PrVP3A1-31N1N2, PrVP3A32-69N1N2, PrVP3A32-61N1N2 and PrMBP proteins were found retained at the cell cytoplasm. Contrarily, nuclear translocation of wild-type and truncated proteins were not observable at 10 hr post-microinjection.

### Apoptotic effect of truncated VP3 protein in MCF cells and Chang cells

**Figure 6:**
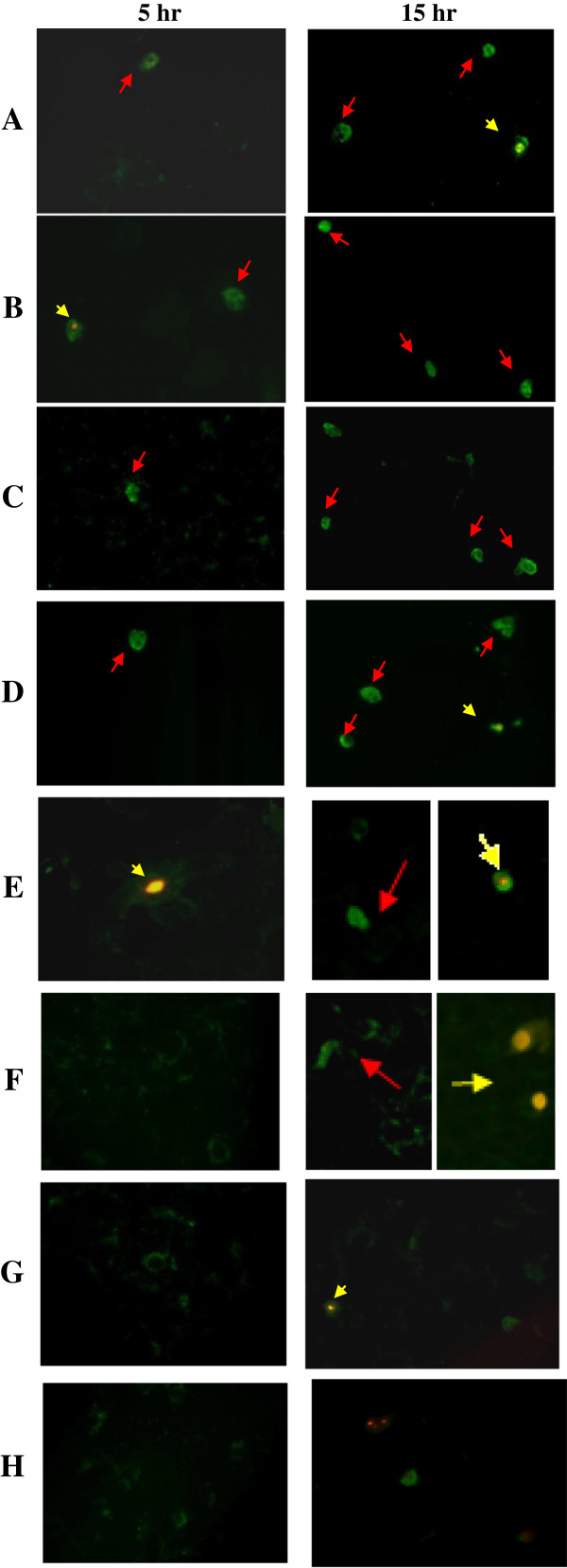
**Detection of Apoptotic MCF7 cells by Annexin V and PI.** MCF7 cells that were microinjected with **A)** PrVP3, **B)** PrVP3A1-69N1N2, **C)** PrVP3A1-46N1N2, **D)** PrVP3A1-31N1N2, **E)** PrVP3A32-69N1N2, **F)** PrVP3A32-62N1N2, **G)** negative control PrMBP and **H)** non-injected cells were analyzed by Annexin V assay at 5 hr and 15 hr. Green fluorescence signal in the cell showed the presence of apoptotic cells (red arrow), on the other hand the presence of both green and red fluorescence in the cell indicated cell necrosis (yellow arrow). Lesser apoptotic cells were observed at 5 hr than 15 hr.

**Figure 7:**
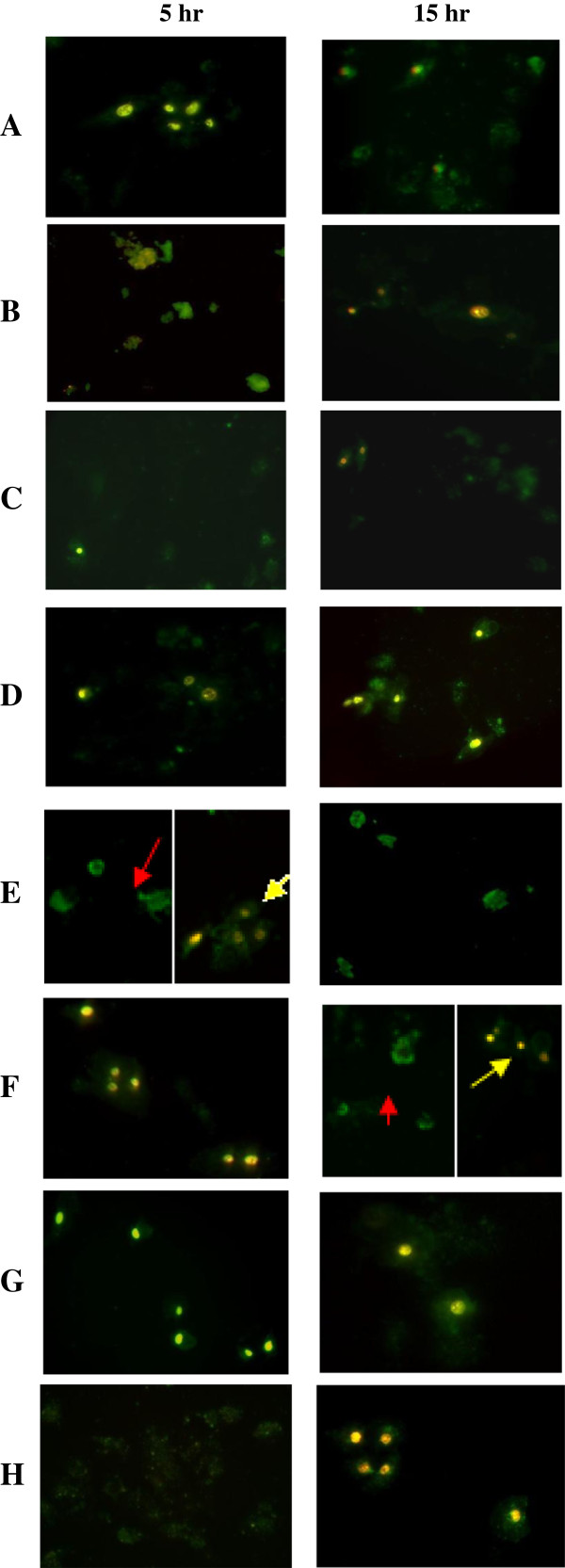
**Apoptosis assay in non-cancerous Chang cells.** Chang cells that were microinjected with **A)** PrVP3, **B)** PrVP3A1-69N1N2, **C)** PrVP3A1-46N1N2, **D)** PrVP3A1-31N1N2, **E)** PrVP3A32-69N1N2, **F)** PrVP3A32-62N1N2, **G)** negative control, PrMBP and **H)** non-injected cells were analyzed by Annexin V assay at 5 hr and 15 hr. Both green and red fluorescence signal in the cell showed the presence of necrotic cells (yellow arrow). No apoptotic cell was detected in **A**-**D** and **G**. Green fluorescence in **E** and **F** showed the presence of apoptotic cells at 15 hr time point (red arrow).

Both MCF7 cells and Chang cells were harvested at various time points to detect the apoptotic cells (Figure 6 and Figure 7). Cells without injection (MCF-7 cells and Chang cells) were either viable or have undergone necrosis showing none or halogreen or both green and red fluorescence signal respectively (Figures 6 and 7). PrVP3, PrVP3A1-69N1N2, PrVP3A1-46N1N2 and PrVP3A1-31N1N2 treatment induced apoptosis in MCF7 cells but not in Chang cells, whilst both PrVP3A32-69N1N2 and PrVP3A32-62N1N2 caused apoptotic effect in both MCF7 cells and Chang cells (Figure 6 and Figure 7).

The recombinant proteins, PrVP3,and PrVP3A1-69N1N2, PrVP3A1-46N1N2,PrVP3A1-31N1N2, PrVP3A32-69N1N2 and PrVP3A32-62N1N2 caused apoptosis in MCF7 at 15 hr post-microinjection as shown in Figure 6. During early hour of post-microinjection (5 hr), a few apoptotic cells were detected in MCF7 cells. However, both PrVP3A32-69N1N2 and PrVP3A32-69N1N2 proteins induced apoptosis in Chang cells (Figure 7).

PrMBP served as the negative control to the recombinant Apoptin protein, where Apoptin protein and truncated Apoptin proteins were fused to the N-terminal of the MBP protein. As expected, PrMBP did not cause any apoptosis effect in both cancerous cells and normal cells. Besides, MBP protein does not have any cytotoxic effect on cancerous cell line nor normal cell line (Figures 6 and 7).

## Discussion

In this study, the wild-type and truncated Apoptins were delivered via cytoplasmic microinjection. To date; needle-microinjection is widely used for the study of many different cell responses in various fields such as cytology, physiology, genetic engineering, molecular biology, virology, tumor biology, developmental biology, pharmacology and toxicology. The injected reagents include cellular organelles, proteins, enzymes, antibodies, genes, metabolites, ions, DNA constructs, RNA, various markers and peptides [18]. This method was of preference over other delivery approach because i) protein with fixed amount could be injected into the cells, ii) an active protein could be injected into the cells instead of transferring the plasmid into the cells for gene activation and protein expression, and iii) a fixed number of cells could be injected to optimize towards a standardized approach [18,19]. Therefore, direct injection of protein to cells was an essential step in our work to predict the outcome of various truncated Apoptins, by passing the possibility of loss of expression when using recombinant plasmid or viral DNA [1,2].

This article described the generation of MBP-tagged truncated CAV-VP3 recombinant proteins and their effect in normal and cancerous cells. Apoptin protein is aggregated, shielded and degraded in the cytoplasm of normal cell [20]. In transformed cells, Apoptin is translocated into the nucleus and caused apoptosis via activation by the tumor-specific pathway [21]. Previous finding by Danen-van Oorschot and colleagues (2003) indicated reduced cell killing activity upon deletion of either N-terminal (a.a. 1–69) or C-terminal (a.a. 70–121) halves of Apoptin. Protein consisting the N-terminal (a.a 1–69) was found in the cytoplasm whereas the C-terminal (a.a 70–121) protein resided in cell nucleus [8]. Although, the NLS signals which shuttle the expressed protein in the nucleus were intact in the C-terminal protein and as anticipated, the protein managed to be translocated into the nucleus, the apoptosis effect was still low (Danen-Van *et al*., [8]). Their finding was further justified by Leliveld and associates [5].

Heilman and coworkers reveal a hydrophobic leucine-rich stretch (LRS) at a.a. 33–46 in N-terminal which acts as self-association region and binding region for promyelocytic leukemia protein and other protein partner [22]. This LRS has influence in nuclear accumulation, whereby deletion or mutation at this region could reduce nuclear accumulation and thus reducing the tumor cell-specificity nuclear targeting signal-dependent nuclear accumulation [10]. In addition to the NLSs which resides at a.a. 82–88 and a.a. 111–121, the C-terminal of Apoptin has a nuclear export sequence (NES) at a.a. 97–105 [7]. NES acts as a nuclear export signal and it is important in contributing to the ability of the Apoptin to be strongly localized in tumor cells instead of normal cells [9]. The C-terminal also consists a phosphorylation site (Thr-108) that assists in driving Apoptin for nuclear accumulation via inactivation of NES in tumor cells (Rohn *et al*., [10]). However, Thr-108 phosphorylation has only partial effect on Apoptin’s apoptotic activity and it is not required for the tumor-specific nuclear localization [11].

In the present study, 5 sets of truncated proteins were derived from the wild-type Apoptin, with each consists a combination of selective regions at N-terminal and NLSs. All the truncated Apoptin proteins contained both NLSs, and the differences between them were in the combination at N-terminal. Apoptin region at a.a. 1–69, a.a. 1–45, a.a. 1–31, a.a. 32–69 and a.a. 32–62 were selected to combine with NLSs respectively with the presence of LRS (a.a. 33–46) and in the absence of NES (a.a. 97–105) and T^108^. Similar to the wild-type Apoptin, all the truncated recombinant proteins were still found at the cytoplasm (injection site) of both cell types at the beginning of injection (2 hr time point). Later in the event at 10 hr post-microinjection, the truncated ApoptinPrVP3A1-69N1N2, PrVP3A1-46N1N2, and PrVP3A1-31N1N2 were detected in the nucleus of MCF7 cells and cytoplasm of Chang cells. This indicated the equivalent ability of those truncated Apoptin to that of wild-type to translocate protein to the nucleus. These truncated proteins retained the ability in inducing apoptotic effect selectively to cancerous cells. Although previous work indicated a limit in apoptotic activity when using either N-terminal or C-terminal (Danen-Van *et al*., [8]), combination of minimal functional sites from both terminals managed to induce apoptosis in MCF-7. Nevertheless, due to the nature of the experiment which required individual protein injection into each cell to standardize the injected protein dose, the number of cells investigated was not permissive for quantitative measurement.

Intriguingly, PrVP3A32-69N1N2 and PrVP3A32-62N1N2 induced apoptosis in both MCF7 cells and Chang cells. Both proteins had deletion at a.a. 1–31, which was an upstream of LRS region. Deletion of this region has led to an unpredictable selectivity of Apoptin. In a previous study, NES linked truncated Apoptin (a.a. 70–121) showed apoptosis in tumor cells but not in normal cells (Danen-von *et al*., [8]). However, the present study demonstrated that the presence of LRS in the absence of NES was sufficient to induce apoptosis in cancerous cells, provided the upstream of LRS (a.a. 1–31) was retained. Previously, a truncated Apoptin lacking LRS and its upstream region (a.a. 1–31), whilst retaining NES, NLS1, NLS2 and T108 regions managed to apoptosize cancer cells selectively (Danen-von *et al*., [8]). Contrarily, this study had proven that cancerous cells selectivity were not totally dependent on the presence of NES. A recent study by Yang and associates showed deletion of a.a 1–30 retained the characteristic nature of Apoptin in the bladder cancer cells [23]. The only significant difference between Yang’s group and ours was the standardized microinjection-mediated-truncated protein delivery versus cell transfection of recombinant plasmid DNAs for expressing truncated proteins within. Another aspect of concern would be the potential induction of apoptosis in normal cells during Apoptin abundance in cytoplasm which might correlate to the nature of transduction [24]. Successfully delivery of Apoptin into cells and efficient expression of Apoptin are essential requirements. Trans-acting activator of transcription (TAT) protein transduction domain (PTD) has been demonstrated to be an ideal agent of delivering Apoptin into cells and *E. coli* plays an important role in expressing full length Apoptin [25].

Trans-acting activator of transcription (TAT) protein transduction domain (PTD) has been demonstrated to be an ideal agent of delivering Apoptin into cells and *E. coli* plays an important role in expressing full length Apoptin [25,26].

## Conclusions

We have demonstrated selective apoptosis induction in MCF-7 cells upon standardized dose administration of PrVP3A1-69N1N2, PrVP3A1-46N1N2, and PrVP3A1-31N1N2. All the 3 truncated proteins consist an upstream region from the known LRS, spanning at a.a. 1–31. The loss of selectivity for MCF-7 cells in both PrVP3A32-69N1N2 and PrVP3A32-62N1N2 could be implicated by the absence of this critical region which strongly suggested as an indispensable domain for target the apoptosis. This study warrants further work towards quantifying the level of apoptosis induced by these truncated proteins, with a linked-within protein-transduction-domain (PTD).

## Abbreviations

a.a: Amino acid; LRS: Hydrophobic leucine-rich stretch; MBP: Maltose binding protein; NLS: Nuclear localization site.

## Competing interests

The authors declare that they have no competing interests.

## Authors’ contributions

LSN carried out the molecular genetic studies and immunoassays, participated in the sequence alignment and drafted the manuscript. ZNA participated in the design of the study, performed the statistical analysis and helped to draft as well as proofread the manuscript. MML, AMO. and FO have supported and facilitated in the research work progress. All the authors have read and approved the final manuscript.

## Pre-publication history

The pre-publication history for this paper can be accessed here:

http://www.biomedcentral.com/1471-2407/13/488/prepub
